# TGF-β1、Smad2和Smad4蛋白表达在可手术切除非小细胞肺癌中的表达

**DOI:** 10.3779/j.issn.1009-3419.2015.09.03

**Published:** 2015-09-20

**Authors:** 勉 谢, 朝生 何, 慎海 魏

**Affiliations:** 510120 广州，广州医科大学附属第一医院呼吸疾病国家重点实验室，广东省人民医院内科 China State Key Laboratory of Respiratory Disease, The First Affiliated Hospital of Guangzhou Medical University, Guangzhou 510120, China

**Keywords:** 转化生长因子β1, Smad, 肺肿瘤, Transforming growth factor β, Smad, Lung neoplasms

## Abstract

**背景与目的:**

转化生长因子β（transforming growth factor β, TGF-β）-Smad信号转导通路中任何一个环节的变化，都会导致信号传递的异常，使细胞生长分化失去控制，从而导致肿瘤发生。本研究旨在探讨可手术切除非小细胞肺癌（non-small cell lung cancer, NSCLC）TGF-β1、Smad2和Smad4的表达与预后的关系。

**方法:**

应用免疫组化方法观察85例NSCLC中TGF-β1、Smad2和Smad4的表达，并分析三种蛋白的表达与临床病理因素和预后的关系。

**结果:**

TGF-β1阳性表达与肺癌分期较晚和淋巴结转移有关。Smad2的表达与各临床病理因素无关。Smad4表达阴性者较Smad4表达阳性者分期晚（*P*=0.014）。多因素分析显示淋巴结转移（*P*=0.001）是85例NSCLC的独立预后因素。47例肺腺癌多因素分析显示TGF-β1（*P*=0.032）和N分期（*P*=0.028）与预后有关。肺腺癌中TGF-β1表达阳性与生存期不良预后有关（*P*=0.0376）。

**结论:**

TGF-β1可能与手术切除后肺腺癌预后有关。

## Abstract (en)

**Background and objective:**

It has been proven that any changes of transforming growth factor β (TGF-β)-Smad signal transduction pathway will lead to abnormalities of signal transmission and the out of control during cell growth and differentiation, resulting in cancer development. The aim of this study is to investigate the prognostic values of TGF-β1, Smad2 and Smad4 in resected non-small cell lung cancer (NSCLC).

**Methods:**

The expression of TGF-β1, Smad2, and Smad4 was evaluated by immunohistochemistry in 85 patients with NSCLC. The relationships among the expression of these proteins, clinicopathological factors, and prognosis were also analyzed.

**Results:**

TGF-β1 positive expression was significantly correlated with the late stage and lymph node involvement. No significant association existed between the expression of Smad2 and the clinicopathological characteristics. The lack of Smad4 expression was associated with the advanced tumor stage (*P*=0.014). Multivariate analysis indicated that lymph node involvement (*P*=0.001) was an independent prognostic factor in the 85 NSCLC patients. The positive expression levels of TGF-β1 (*P*=0.032) and N stage (*P*=0.028) were demonstrated to be independent risk factors for survival among 47 lung adenocarcinoma patients. Adenocarcinoma patients with TGF-β1 positive expression demonstrated an unfavorable survival outcome (*P*=0.0376).

**Conclusion:**

TGF-β1 may be an independent predictor of survival in resected lung adenocarcinoma patients.

转化生长因子β（transforming growth factor β, TGF-β）是一类具有多种生物学功能的多肽类生长因子超家族，目前已发现5种TGF-β亚型，在哺乳类动物中有TGF-β1、TGF-β2和TGF-β3三种亚型，其中，TGF-β1是人体中发现最多的一种亚型。TGF-β对肺癌具有双重作用。肺癌早期TGF-β信号通路中各个成分改变都将影响TGF-β对肺癌的抑制作用，促进肺癌的发生。在肺癌晚期，由于TGF-β具备的其他功能，如刺激肿瘤血管生长，降低细胞间的粘附以及合成细胞外基质等，为肺癌的生长、转移提供微环境，使肺癌更具侵袭性。

Smads蛋白可分为三类：第一类是受体活化Smads（receptor-activited Smads, R-Smads），包括Smad1、Smad2、Smad3、Smad5和Smad8；第二类是共有Smad（Co-Smad），即Smad4，他是TGF-β超家族信号转导途径中必不可少的调控转录的关键因子。第三类被称为抑制性Smad（inhibitory Smads, I-Smads），包括Smad6和Smad7。Smads蛋白作为受体后下游信号传递分子，其失活或突变同样可导致TGF-β对肺癌生长抑制作用的丧失。

我们前期实验研究（Western blot）发现8株肺癌细胞株的TGF-β1、Smad2蛋白表达较正常支气管上皮细胞增高，Smad4蛋白表达较正常支气管上皮细胞下降，而其他Smads蛋白表达无明显差异（结果未发表）。本研究拟采用免疫组化方法观察非小细胞肺癌（non-small cell lung cancer, NSCLC）中TGF-β1、Smad2、Smad4蛋白的表达，以揭示TGF-β1/Smad信号传导通路在肺癌中的变化，探讨TGF-β1/Smad在肺癌发生发展中的相关机制。

## 1 材料与方法

### 1.1 临床资料

连续选取我院胸外科2003年1月-2003年12月85例行根治性肺叶切除加纵隔淋巴结清扫的初诊肺癌患者；排除存在第二肿瘤患者。其中男性61例，女性24例；年龄34岁-77岁，中位年龄60岁。47例腺癌，28例鳞癌，10例腺鳞癌；Ⅰ期25例，Ⅱ期22例，Ⅲa期38例，淋巴结转移53例，无淋巴结转移32例；术后辅助化疗患者共66例，Ⅰ期13例，Ⅱ期20例，Ⅲa期33例；2例Ⅲa期患者接受术后辅助放疗。患者复发或转移后接受规范化放疗或靶向治疗。患者随访至2009年4月1日。总生存期定义为首次手术至死亡或末次随访时间。随访5年以上，生存期4个月-74个月，83例患者完成随访，2例患者失访。中位生存期36个月，尚存活17人。

### 1.2 免疫组化试验

#### 1.2.1 试剂

兔抗人TGF-β1多克隆抗体（sc-146），稀释度为1:400；兔抗人p-Smad2/3多克隆抗体（sc-11769-R），稀释度为1:400；兔抗人Smad4多克隆抗体（sc-7154），稀释度为1:100，以上试剂购自Santa Cruz公司。辣根酶标记羊抗兔多聚体（pv-6001）工作液试剂盒购自北京中山公司。

#### 1.2.2 试验步骤

依据说明书以TGF-β1阳性的乳腺癌，Smad2、Smad4阳性的胰腺癌分别为TGF-β1、Smad2、Smad4的阳性对照，用PBS缓冲液作为阴性对照，采用sp三步法进行免疫组化检测。具体步骤如下：组织标本常规石蜡包埋，制备4 μm切片，贴附于APES处理的载破片上，65 ℃烘烤1 h，二甲苯脱蜡，梯度乙醇脱水，3%H_2_O_2_室温孵育10 min，以消除内源性过氧化物酶的活性；蒸馏水冲洗，PBS浸泡5 min，置于抗原修复液中微波炉抗原修复，使温度保持92 ℃-98 ℃持续10 min-15 min，室温冷却20 min-30 min使蛋白恢复原有空间构型，PBS冲洗5 min×4次，滴加山羊血清封闭以消除电荷吸附所造成的非特异性背景染色，室温放置30 min；加稀释的一抗，4 ℃孵育过夜；PBS冲洗5 min×4次；滴加生物素标记二抗，37 ℃孵育30 min，PBS冲洗5 min×4次；滴加辣根酶标记链霉卵白素，37 ℃孵育30 min，PBS冲洗5 ℃×4次；DAB显色，自来水冲洗，复染苏木素，1%盐酸酒精分化，封片。

#### 1.2.3 免疫组化结果的判断

每张切片随机取3个高倍视野，着色部位明显高于背景或背景不着色而细胞着色者为阳性细胞染色，以阳性肿瘤细胞比例的平均值定义为该肿瘤阳性细胞百分比。TGF-β1结果判断参照Huang等^[1]^的标准：阴性为无着色癌细胞（-）；弱阳性为着色癌细胞比例小于25%（+）；中度阳性为着色癌细胞比例为25%-75%（++）；强阳性为着色癌细胞比例大于75%（+++）。Smad2、Smad4结果判断参照Yang等^[2]^的标准：阴性为无着色癌细胞（-）；阳性为着色癌细胞比例为5%-30%（+）；强阳性为着色癌细胞比例大于30%（++）。以癌旁间质细胞中出现同上染色颗粒为癌旁组织阳性表达。

### 1.3 统计学分析

采用统计学软件SPSS 16.0进行数据分析，采用*Pearson*卡方、校正卡方检验或*Fisher’s*精确检验。*Logistic*回归分析TGF-β1、Smad2、Smad4、P分期、N分期、T分期作为独立变量对总生存期的预测影响；首先行单因素*Cox*和*Kaplan-Meier*分析，多因素分析采用*Cox*比例风险回归模型。*Log-rank*检验评价单因素生存分析结果。*P* < 0.05为差异有统计学意义。

## 2 结果

### 2.1 TGF-β1的表达与临床病理的关系

TGF-β1主要定位于肿瘤细胞，阳性结果为细胞胞浆呈棕褐色（图 1A）。85例肺癌组织中，TGF-β1表达阴性为37例（43.53%），TGF-β1表达弱阳性为13例（15.29%），TGF-β1表达中度阳性为15例（17.65%），TGF-β1表达强阳性为20例（23.53%）。淋巴结转移组（N1和N2）的TGF-β1表达率明显高于无淋巴结转移组（N0）（*P*=0.002）。Ⅲ期肺癌TGF-β1表达明显高于Ⅰ期、Ⅱ期（*P* < 0.001）。TGF-β1的表达与患者年龄、性别、组织类型以及远处转移复发无关（表 1）。

**1:**
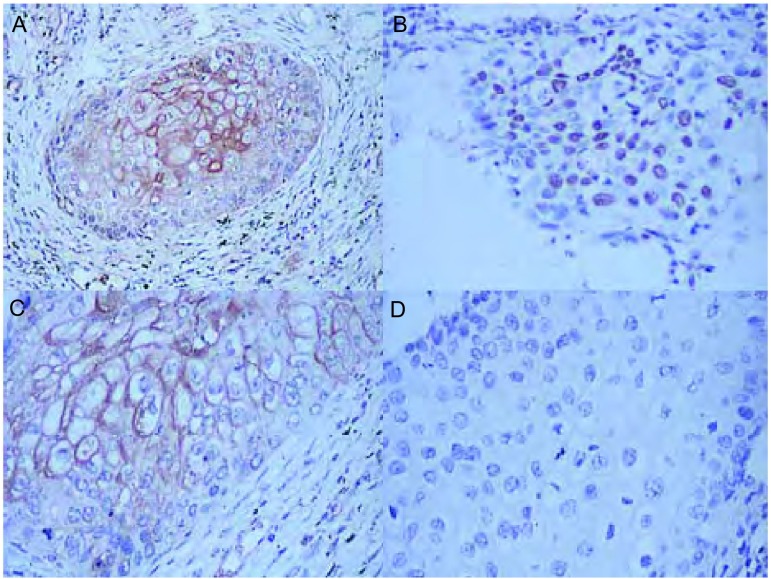
非小细胞肺癌TGF-*β*1、Smad2和Smad4的表达。A：肺鳞癌表达TGF-*β*1，呈细胞浆阳性（SP，×200）；B：肺腺癌表达Smad2，呈细胞核阳性（SP，×200）；C：肺鳞癌表达Smad4，呈细胞浆阳性（SP，×400）；D：阴性对照（SP，×400） TGF-*β*1, Smad2, and Smad4 expression in non-small cell lung cancer. A: TGF-*β*1 expression in lung squamous carcinoma (cellular localization in cytoplasm) (×200); B: Smad2 expression in lung adenocarcinoma (cellular localization in nucleus) (×200); C: Smad4 expression in lung squamous carcinoma (cellular localization in cytoplasm) (×400); D: Negative control (×400)

**1:** 85例非小细胞肺癌患者的TGF-*β*1、Smad2和Smad4表达与临床病理指标的关系 The correlationship between the expressions of TGF-*β*1, Smad2, Smad4 and clinicopathological characteristics in 85 NSCLC patients

Charcteristic		TGF-*β*1		*P*	Smad2		*P*	Smad4		*P*
		Negative	Positive		Negative	Positive		Negative	Positive	
Age (year)	≥60	23	29	0.870	3	49	0.526	6	46	> 0.999
	< 60	14	19		4	29		3	30	
Gender	Male	24	37	0.234	4	57	0.646	4	57	0.125
	Female	13	11		3	21		5	19	
ECOG PS	0-1	28	38	0.568	5	62	0.217	7	66	0.351
	2	9	10		2	16		2	10	
Smoke history	Yes	21	29	0.625	4	46	0.407	5	42	0.398
	No	16	19		3	32		4	34	
Pathologic stage	Ⅰ-Ⅱ	34	13	< 0.01	5	42	0.617	1	46	0.014
	Ⅲa	13	25		2	36		8	30	
T stage	T1	2	4	0.654	2	4	0.120	2	4	0.199
	T2	18	27		3	42		4	41	
	T3	17	17		2	32		3	31	
N stage	N0	21	11	0.002	4	28	0.481	2	30	0.518
	N1-2	16	37		3	50		7	46	
Differentiation	Well	10	8	0.436	2	16	0.598	3	15	0.475
	Moderate	12	15		3	24		3	24	
	Poor	15	25		2	38		3	37	
Histology	Adenocarcinoma	19	28	0.198	4	43	> 0.999	5	42	0.467
	Squamous	11	17		2	26		2	26	
	Adenosquamous	7	3		1	9		2	8	
Metastasis	Yes	31	39	0.129	6	70	> 0.999	8	70	0.186
	No	6	9		1	8		1	6	
5-yr survival	PFS (%)	27.2	18.8	0.124	19.8	25.5	0.212	24.6	15.6	0.315
	OS (%)	51.6	25.7	0.003	26.8	48.9	0.139	50.1	25.0	0.267
PFS: progression-free survival; OS: overall survival; ECOG: Eastern Cooperative Oncology Group; PS: performance status; NSCLC: non-small cell lung cancer; TGF-*β*1: transforming growth factor *β*1.										

### 2.2 Smad2、Smad4的表达与临床病理的关系

Smad2主要定位于肿瘤细胞，阳性结果为细胞核呈棕褐色（图 1B）。Smad2表达阳性78例（91.76%）。Smad2的表达与各临床病理因素无关（表 1）。Smad4主要定位于肿瘤细胞，阳性结果为细胞胞浆呈棕褐色（图 1C）。Smad4表达阳性76例（89.41%）。Smad4表达阴性者肺癌分期较晚（*P*=0.014）（表 1）。

### 2.3 TGF-β1、Smad2、Smad4的表达与生存期的关系

TGF-β1表达阴性组的生存期与TGF-β1表达阳性组比较无统计学差异（*P*=0.294）（图 2A）。肺腺癌中，TGF-β1表达阴性组生存期长于TGF-β1表达阳性性组（*P*=0.038）（图 2B）。TGF-β1表达阳性组5年总生存率为25.7%；TGF-β1表达阴性组为51.6%，较TGF-β1表达阳性组明显增加（*P*=0.003）（表 1）。TGF-β1表达阴性组和阳性组5年无病生存率无明显差异（*P*=0.124）。Smad2、Smad4的表达与5年PFS率无关。Smad2和Smad4的表达与生存期无关（Smad2 *P*=0.409；Smad4 *P*=0.596）（图 3）。85例肺癌单因素分析显示淋巴结转移（*P*=0.001）和肺癌分期（*P*=0.001）与预后有关，多因素分析显示淋巴结转移（*P*=0.001）为独立的预后因素（表 2）。47例肺腺癌多因素分析显示TGF-β1（*P*=0.032）和N分期（*P*=0.028）为独立的预后因素（表 3）。

**2:**
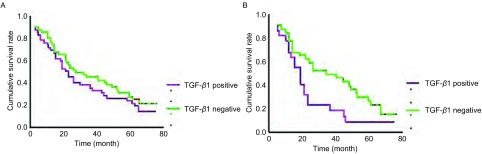
NSCLC患者TGF-*β*1表达阴性组和阳性组的生存曲线。A：NSCLC患者TGF-*β*1表达阴性组和阳性组的生存曲线；B：肺腺癌患者TGF-*β*1表达阴性组和阳性组的生存曲线 Influence of TGF-*β*1 expression on overall survival. A: Influence of TGF-*β*1 expression on overall survival in NSCLC patients (*Kaplan-Meier* multivariate analysis); B: Influence of TGF-*β*1 expression on overall survival in lung adenocarcinoma patients (*Kaplan-Meier* multivariate analysis)

**3:**
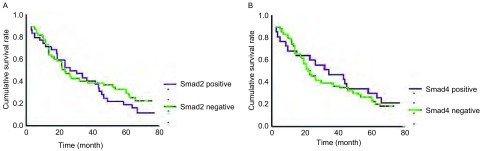
NSCLC患者Smad2和Smad4表达阴性组和阳性组的生存曲线。A：NSCLC患者Smad2表达阴性组和阳性组的生存曲线；B：NSCLC患者Smad4表达阴性组和阳性组的生存曲线 Influence of Smad2 and Smad4 expression on overall survival. A : Influence of Smad2 expression on overall survival in NSCLC patients (*Kaplan-Meier* multivariate analysis); B: Influence of Smad4 expression on overall survival in NSCLC patients (*Kaplan-Meier* multivariate analysis)

**2:** 85例非小细胞肺癌患者总生存期的多因素分析 *Kaplan-Meier* multivariate survival analysis in 85 NSCLC patients

Variable	HR (hazard ratio)	95%CI	*P*
TGF-*β*1	1.398	0.822-2.378	0.216
Smad2	1.047	0.537-2.038	0.894
Smad4	0.677	0.334-1.370	0.278
Pathologic tumor stage	0.666	0.383-1.158	0.150
N stage	2.415	1.420-4.105	0.001
T stage	0.677	0.396-1.155	0.153

**3:** 47例肺腺癌患者总生存期的多因素分析 *Kaplan-Meier* multivariate survival analysis in 47 adenocarcinoma patients

Variable	HR	95%CI	*P*
T stage	0.634	0.318-1.263	0.195
N stage	0.335	0.126-0.890	0.028
Pathologic tumor stage	2.008	0.915-5.899	0.062
TGF-*β*1	2.126	1.069-4.228	0.032
Smad2	2.353	0.922-6.007	0.074
Smad4	1.478	0.674-3.242	0.330

## 3 讨论

TGF-β/Smad信号转导途径在参与肿瘤形成过程中发挥重要作用。TGF-β在肿瘤细胞中过度表达不仅通过刺激血管发生及其潜在的免疫抑制作用促进肿瘤发展，而且能够直接影响肿瘤细胞的浸润和转移。TGF-β对肿瘤的直接影响可以通过Smads依赖途径或干扰Smads依赖途径介导完成。TGF-β1能诱导c-sis的表达，但抑制c-myc的表达，这种诱导或抑制作用与作用细胞种类及TGF-β的不同功能有关。TGF-β1的自分泌作用在较晚期的肿瘤中受到抑制，减弱了对原癌基因*c-myc*的抑制作用，加速肿瘤细胞从G_1_期进入S期，促进肿瘤细胞的增殖^[3]^。我们的研究表明TGF-β1阳性表达与肺癌分期较晚和淋巴结转移有关，85例NSCLC中TGF-β1表达阳性率为56.47%，Ⅲ期肺癌TGF-β1表达明显高于Ⅰ期、Ⅱ期（*P* < 0.001）。Hasegawa等^[4]^采用酶联免疫吸附方法实验亦发现Ⅲ期NSCLC患者TGF-β1蛋白水平明显高于Ⅰ期、Ⅱ期的患者。

Smad2是TGFβ抗增殖应答和调节转录的重要级联分子，是TGFβ-Smad信号通路中关键转导分子之一，其生物活性改变将会影响通路功能，引起细胞周期或细胞表型的改变^[5-7]^。在TGFβ-Smad信号通路中任一因子失活均可干扰TGFβ-Smad信号转导通路功能，导致细胞生物行为变化。本研究结果显示在NSCLC中Smad2表达阳性者分期相对较早，但差异无统计学意义，Smad4表达阳性者肺癌分期较早（*P*=0.009），说明Smad蛋白可能充当肿瘤抑制因子，另外一些癌基因蛋白通过与Smad蛋白相作用抑制Smad蛋白的功能^[8]^。肿瘤细胞可以选择性逃逸信号诱导的增长抑制或凋亡反应，TGF-β1这些对肿瘤的直接影响都是通过Smads蛋白自主的途径所介导，或通过干扰Smads依赖的途径而作用的。TGF-β1介导的生长抑制功能可被显性失活的Smad2、Smad3或Smad4全部抑制。Nye等^[7]^考察了人肺癌中分离到的6个Smad2和Smad4突变子的生物学和生物化学功能，发现由于不能同野生型Smads形成同源和（或）异源寡聚物，Smad2和Smad4的所有突变体均在TGF-β转导的转化生长抑制信号中缺失，且不能活化由Smad/hFSAT-1介导的转录，结果提示Smads突变体之间不能形成功能性配对，这些异常可能在肿瘤发生中起作用。

目前尚无文献报道SMAD蛋白的表达与肺癌预后的关系。本研究中多因素分析显示Smad2和Smad4表达与生存期无关，而TGF-β1是可切除肺腺癌术后独立预后因素。Liu等^[9]^发现TGF-β1与NSCLC分化差有关，影响肺癌进展和转移，可作为NSCLC预后指标之一。Yang等^[10]^发现肺腺癌高TGF-β1表达组（≥289 pg/mL）比低TGF-β1表达组（< 289 pg/mL）的总生存期短。Takanami等^[11]^用免疫组化方法检测120例肺腺癌组织TGF-β1的表达，结果显示TGF-β1阴性表达组生存期比阳性表达组延长，对96例完全切除的肺腺癌病例行*Cox*回归分析表明TGF-β1表达水平和肺癌分期是生存期的独立预后因素。TGF-β1在肿瘤中可诱导表达VEGF、金属蛋白酶（matrix metallopeptidase 2, MMP-2）和MMP-9，负调控MMP抑制因子，提供蛋白酶丰富的微环境，有利于肿瘤细胞的迁移和浸润适当的血管上皮细胞^[12]^。肿瘤细胞分泌的TGF-β1能帮助瘤细胞逃避宿主免疫系统的监控，易化侵袭和转移过程^[13]^。TGF-β1是细胞外基质产生和沉积的重要调节因子。体外试验^[14]^发现TGF-β1通过调控内皮细胞的基因表达，通过增加包括胶原蛋白、纤维蛋白在内的细胞外基质的形成和聚集导致了肺腺癌中心纤维化的形成，并且也发现肺腺癌中心纤维化的形成与肺腺癌的预后有关。

TGF-β1/Smads在肺癌中与其他信号系统如BMP/BMPR2和Wnt/β-catenin等存在相互交联，我们仍需行进一步基础研究阐述TGF-β在肺癌中的详细作用机制。本研究是回顾性单纯病例研究，只选择能根治性切除的NSCLC作为研究对象，虽然患者在复发转移后均接受规范化放疗或靶向治疗，但是并非所有患者在病情进展的后续治疗仍存在异质性；因此本研究存在一定的偏倚。关于肺癌手术后复发转移后多种治疗方式的先后顺序存在争议^[16]^，是否影响总生存期尚不确定。我们将扩大研究病例群体，进一步验证本研究的初步结果。
